# Bis[4-(methyl­amino)benzoato-κ*O*]bis­(nicotinamide-κ*N*)zinc(II)

**DOI:** 10.1107/S1600536808042852

**Published:** 2008-12-20

**Authors:** Barış Tercan, Tuncer Hökelek, Özgür Aybirdi, Hacali Necefoğlu

**Affiliations:** a Karabük University, Department of Physics, 78050 Karabük, Turkey; b Hacettepe University, Department of Physics, 06800 Beytepe, Ankara, Turkey; c Kafkas University, Department of Chemistry, 63100 Kars, Turkey

## Abstract

The title zinc complex, [Zn(C_8_H_8_NO_2_)_2_(C_6_H_6_N_2_O)_2_], is composed of two monodentate 4-(methyl­amino)benzoate and two monodentate nicotinamide ligands. The coordination around the Zn atom is distorted tetra­hedral. The dihedral angles between the two benzene rings and the two pyridine rings are 78.30 (6) and 68.86 (5)°. In the crystal structure, inter­molecular N—H⋯O hydrogen bonds link the mol­ecules into an infinite three-dimensional network.

## Related literature

For general backgroud, see: Antolini *et al.* (1982 ▶); Krishnamachari (1974 ▶); Nadzhafov *et al.* (1981 ▶). For related structures, see: Necefoğlu *et al.* (2002 ▶); Hökelek *et al.* (2007 ▶).
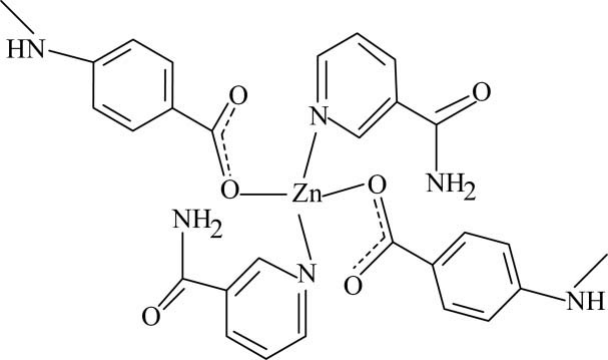


## Experimental

### 

#### Crystal data

[Zn(C_8_H_8_NO_2_)_2_(C_6_H_6_N_2_O)_2_]
*M* *_r_* = 609.93
Monoclinic, 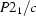

*a* = 8.085 (4) Å
*b* = 16.036 (7) Å
*c* = 21.333 (4) Å
β = 95.78 (3)°
*V* = 2751.8 (19) Å^3^
*Z* = 4
Mo *K*α radiation
μ = 0.95 mm^−1^
*T* = 294 (2) K
0.55 × 0.20 × 0.10 mm

#### Data collection

Rigaku AFC-7S diffractometer
Absorption correction: ψ scan (North *et al.*, 1968 ▶) *T*_min_ = 0.624, *T*_max_ = 0.911
17939 measured reflections
17079 independent reflections
7521 reflections with *I* > 2σ(*I*)
*R*_int_ = 0.045
3 standard reflections every 150 reflections intensity decay: 1%

#### Refinement

*R*[*F* ^2^ > 2σ(*F* ^2^)] = 0.044
*wR*(*F* ^2^) = 0.148
*S* = 1.00
17079 reflections
380 parameters
H atoms treated by a mixture of independent and constrained refinement
Δρ_max_ = 0.67 e Å^−3^
Δρ_min_ = −0.58 e Å^−3^

### 

Data collection: *MSC/AFC Diffractometer Control Software* (Molecular Structure Corporation, 1994 ▶); cell refinement: *MSC/AFC Diffractometer Control Software*; data reduction: *TEXSAN for Windows* (Molecular Structure Corporation, 1997 ▶); program(s) used to solve structure: *SHELXS97* (Sheldrick, 2008 ▶); program(s) used to refine structure: *SHELXL97* (Sheldrick, 2008 ▶); molecular graphics: *ORTEP-3* (Farrugia, 1997 ▶) and *Mercury* (Macrae, 2006 ▶); software used to prepare material for publication: *WinGX* (Farrugia, 1999 ▶).

## Supplementary Material

Crystal structure: contains datablocks I, global. DOI: 10.1107/S1600536808042852/su2084sup1.cif

Structure factors: contains datablocks I. DOI: 10.1107/S1600536808042852/su2084Isup2.hkl

Additional supplementary materials:  crystallographic information; 3D view; checkCIF report

## Figures and Tables

**Table:** 

Zn1—O1	1.9584 (14)
Zn1—O3	1.9210 (14)
Zn1—N1	2.0722 (15)
Zn1—N3	2.0854 (15)

**Table:** 

O1—Zn1—N1	96.85 (6)
O1—Zn1—N3	105.85 (6)
O3—Zn1—O1	143.41 (6)
O3—Zn1—N1	105.91 (5)
O3—Zn1—N3	95.81 (6)
N1—Zn1—N3	104.58 (5)

**Table 2:** Hydrogen-bond geometry (Å, °)

*D*—H⋯*A*	*D*—H	H⋯*A*	*D*⋯*A*	*D*—H⋯*A*
N2—H2*B*⋯O2^i^	0.86	2.00	2.839 (3)	166
N4—H4*B*⋯O6^ii^	0.86	2.12	2.950 (3)	161
N6—H61⋯O4^ii^	0.88 (2)	2.10 (2)	2.955 (3)	166 (2)

Symmetry codes: (i) 
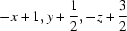
; (ii) 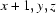.

**Fig. 1.**
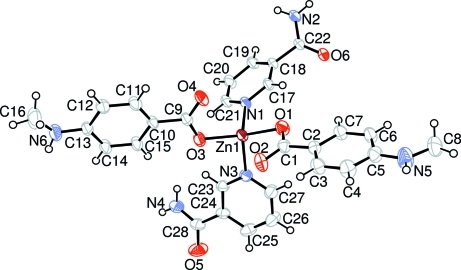
The molecular structure of the title complex, with the atom-numbering scheme and displacement ellipsoids drawn at the 40% probability level.

**Fig. 2.**
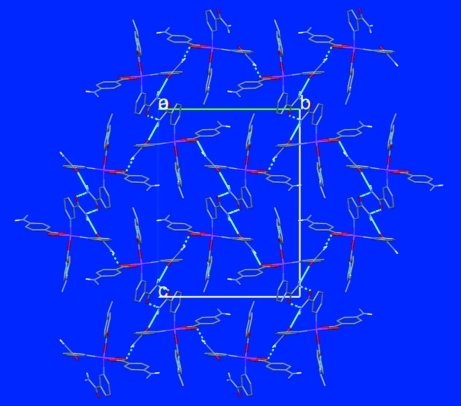
A view down the a axis of the crystal packing of the title compound, showing the N-H···O hydrogen bonds (blue dashed lines) linking the molecules to form an infinite three-dimensional network (H atoms not involved in hydrogen bonding have been omitted for clarity).

## supplementary crystallographic information

### Comment

Transition metal complexes with biochemical molecules show interesting physical and/or chemical properties, through which they may find applications in biological systems (Antolini *et al.*, 1982). The structure-function-coordination relationships of the arylcarboxylate ion in Zn^II^ complexes of benzoic acid derivatives may be changed, depending on the nature and position of the substituted groups on the benzene ring, the nature of the additional ligand molecule or solvent, and the medium of the synthesis (Nadzhafov *et al.*, 1981). Nicotinamide (NA) is one form of niacin, and a deficiency of this vitamin leads to loss of copper from the body, known as pellagra disease. Victims of pellagra show unusually high serum and urinary copper levels (Krishnamachari, 1974).

The structure determination of the title compound, a zinc complex with two methylaminobenzoate (MAB) and two nicotinamide (NA) ligands, was undertaken in order to determine the properties of the MAB and NA ligands and also to compare the results obtained with those reported previously.

The molecular structure of the title monomeric complex, containing two monodenate MAB and two monodentate NA ligands, is illustrated in Fig. 1. The coordination around the Zn atom is distorted tetrahedral (Table 1).

The near equalities of the C1—O1 [1.284 (2) Å], C1—O2 [1.248 (2) Å] and C9—O3 [1.278 (2) Å], C9—O4 [1.241 (2) Å] bonds in the carboxylate groups indicate delocalized bonding arrangements, rather than localized single and double bonds, as in the other zinc(II) complexes: bis(4-hydroxybenzoato-κ*O*)bis(nicotinamide-κ*N*)zinc(II) (Necefoğlu *et al.*, 2002) and diaquabis(*N*,*N*'-diethylnicotinamide-κ*N*)bis(4-\ fluorobenzoato-κ*O*)- zinc(II) (Hökelek *et al.*, 2007). This may be due to the intramolecular N—H···O hydrogen bonding of the carboxylate O atoms (Table 2).

The Zn atom is displaced out of the least-squares plane of the carboxylate groups (O1/C1/O2) and (O3/C9/O4) by -0.1070 (3) Å and -0.0967 (4) Å, respectively. The dihedral angles between the planar carboxylate groups (O1/C1/O2) and (O3/C9/O4) and the adjacent benzene rings, A (C2—C7) and B (C10—C15), are 7.92 (13)° and 6.60 (13)°, respectively. The dihedral angles between the two benzene rings, A and B, and the two pyridine rings, C (N1/C17—C21) and D (N3/C23—C27), are A/B = 78.30 (6)° and C/D = 68.86 (5)°. The benzene rings are oriented with respect to the pyridine rings at dihedral angles of A/C = 20.95 (5)°, A/D = 77.94 (6)°, B/C = 80.59 (5)° and B/D = 33.66 (6)°.

As can be seen from the crystal packing diagram (Fig. 2), the molecules are linked by intermolecular N—H···O hydrogen bonds to form an infinite three-dimensional network (Table 2).

### Experimental

The title compound was prepared by the reaction of ZnNO_4_ (1.61 g, 10 mmol) in H_2_O (25 ml) and nicotinamide (2.44 g, 20 mmol) in H_2_O (100 ml), with sodium 4-*N*-methylaminobenzoate (3.00 g, 20 mmol) in H_2_O (100 ml), at room temperature. The mixture was filtered and set aside to crystallize at ambient temperature for one week, giving colorless single crystals.

### Refinement

The NH H-atoms, H51 and H61, were located in difference Fourier maps and freely refined [N—H = 0.88 (2) and 0.90 (3) Å, resepectively]. The remaining H atoms were positioned geometrically and treated as riding atoms: N-H = 0.86 Å, C-H = 0.93 - 0.96 Å, with U_iso_(H) = 1.2 or 1.5U_eq_(parent N- or C-atom).

### Crystal data

**Table:**

[Zn(C_8_H_8_NO_2_)_2_(C_6_H_6_N_2_O)_2_]	*F*(000) = 1264
*M**_r_* = 609.93	*D*_x_ = 1.472 Mg m^−^^3^
Monoclinic, *P*2_1_/*c*	Mo *K*α radiation, λ = 0.71073 Å
Hall symbol: -P 2ybc	Cell parameters from 25 reflections
*a* = 8.085 (4) Å	θ = 20.1–27.1°
*b* = 16.036 (7) Å	µ = 0.95 mm^−^^1^
*c* = 21.333 (4) Å	*T* = 294 K
β = 95.78 (3)°	Plate, colorless
*V* = 2751.8 (19) Å^3^	0.55 × 0.20 × 0.10 mm
*Z* = 4	

### Data collection

**Table:**

Rigaku AFC-7S diffractometer	7521 reflections with *I* > 2σ(*I*)
Radiation source: fine-focus sealed tube	*R*_int_ = 0.045
graphite	θ_max_ = 40.0°, θ_min_ = 2.3°
ω/2θ scans	*h* = 0→14
Absorption correction: ψ scan (North *et al.*, 1968)	*k* = 0→29
*T*_min_ = 0.624, *T*_max_ = 0.911	*l* = −38→38
17939 measured reflections	3 standard reflections every 150 reflections
17079 independent reflections	intensity decay: 1%

### Refinement

**Table:**

Refinement on *F*^2^	Primary atom site location: structure-invariant direct methods
Least-squares matrix: full	Secondary atom site location: difference Fourier map
*R*[*F*^2^ > 2σ(*F*^2^)] = 0.044	Hydrogen site location: inferred from neighbouring sites
*wR*(*F*^2^) = 0.148	H atoms treated by a mixture of independent and constrained refinement
*S* = 1.00	*w* = 1/[σ^2^(*F*_o_^2^) + (0.064*P*)^2^] where *P* = (*F*_o_^2^ + 2*F*_c_^2^)/3
17079 reflections	(Δ/σ)_max_ = 0.003
380 parameters	Δρ_max_ = 0.67 e Å^−^^3^
0 restraints	Δρ_min_ = −0.58 e Å^−^^3^

### Special details

**Table:**

Geometry. All e.s.d.'s (except the e.s.d. in the dihedral angle between two l.s. planes) are estimated using the full covariance matrix. The cell e.s.d.'s are taken into account individually in the estimation of e.s.d.'s in distances, angles and torsion angles; correlations between e.s.d.'s in cell parameters are only used when they are defined by crystal symmetry. An approximate (isotropic) treatment of cell e.s.d.'s is used for estimating e.s.d.'s involving l.s. planes.
Refinement. Refinement of *F*^2^ against ALL reflections. The weighted *R*-factor *wR* and goodness of fit *S* are based on *F*^2^, conventional *R*-factors *R* are based on *F*, with *F* set to zero for negative *F*^2^. The threshold expression of *F*^2^ > σ(*F*^2^) is used only for calculating *R*-factors(gt) *etc*. and is not relevant to the choice of reflections for refinement. *R*-factors based on *F*^2^ are statistically about twice as large as those based on *F*, and *R*- factors based on ALL data will be even larger.

### Fractional atomic coordinates and isotropic or equivalent isotropic displacement parameters (Å^2^)

**Table:**

	*x*	*y*	*z*	*U*_iso_*/*U*_eq_	
Zn1	0.70261 (2)	0.883707 (11)	0.824978 (8)	0.02982 (5)	
O1	0.48643 (15)	0.83449 (8)	0.83709 (6)	0.0433 (3)	
O2	0.65060 (16)	0.72567 (9)	0.83365 (7)	0.0499 (3)	
O3	0.89177 (14)	0.87348 (8)	0.77778 (6)	0.0399 (3)	
O4	0.72134 (14)	0.85533 (10)	0.69085 (7)	0.0480 (3)	
O5	1.3622 (2)	0.92075 (12)	1.03306 (7)	0.0696 (5)	
O6	0.12579 (14)	1.06871 (10)	0.83276 (6)	0.0501 (3)	
N1	0.62073 (14)	1.00559 (8)	0.81250 (6)	0.0294 (2)	
N2	0.14054 (17)	1.16197 (10)	0.75439 (8)	0.0461 (4)	
H2A	0.0346	1.1691	0.7494	0.055*	
H2B	0.2033	1.1889	0.7312	0.055*	
N3	0.83802 (17)	0.88321 (8)	0.91350 (6)	0.0332 (3)	
N4	1.31932 (19)	0.98898 (11)	0.94118 (7)	0.0474 (4)	
H4A	1.4181	1.0095	0.9451	0.057*	
H4B	1.2521	1.0007	0.9085	0.057*	
N5	−0.0098 (3)	0.54968 (13)	0.91042 (12)	0.0686 (6)	
H51	0.006 (3)	0.4943 (18)	0.9059 (13)	0.081 (9)*	
N6	1.44462 (19)	0.85451 (14)	0.58922 (8)	0.0509 (4)	
H61	1.538 (3)	0.8568 (15)	0.6141 (11)	0.056 (7)*	
C1	0.51107 (19)	0.75548 (11)	0.84074 (7)	0.0343 (3)	
C2	0.37136 (19)	0.70213 (10)	0.85572 (7)	0.0323 (3)	
C3	0.3855 (2)	0.61500 (11)	0.85629 (9)	0.0419 (4)	
H3	0.4828	0.5904	0.8454	0.050*	
C4	0.2581 (2)	0.56531 (11)	0.87261 (10)	0.0487 (4)	
H4	0.2692	0.5076	0.8713	0.058*	
C5	0.1117 (2)	0.60028 (11)	0.89119 (9)	0.0430 (4)	
C6	0.0946 (2)	0.68736 (11)	0.88884 (9)	0.0418 (4)	
H6	−0.0029	0.7123	0.8992	0.050*	
C7	0.2234 (2)	0.73610 (10)	0.87094 (9)	0.0373 (3)	
H7	0.2099	0.7937	0.8691	0.045*	
C8	−0.1605 (3)	0.57827 (18)	0.93347 (14)	0.0713 (7)	
H8A	−0.1340	0.6133	0.9696	0.107*	
H8B	−0.2241	0.5312	0.9452	0.107*	
H8C	−0.2243	0.6095	0.9011	0.107*	
C9	0.86333 (18)	0.86285 (10)	0.71831 (8)	0.0315 (3)	
C10	1.01355 (17)	0.85983 (10)	0.68309 (7)	0.0298 (3)	
C11	1.00398 (19)	0.84079 (11)	0.61932 (8)	0.0373 (3)	
H11	0.9007	0.8295	0.5977	0.045*	
C12	1.1442 (2)	0.83816 (12)	0.58699 (7)	0.0391 (4)	
H12	1.1342	0.8246	0.5444	0.047*	
C13	1.30180 (19)	0.85588 (11)	0.61844 (7)	0.0342 (3)	
C14	1.31028 (18)	0.87565 (12)	0.68256 (8)	0.0384 (4)	
H14	1.4128	0.8882	0.7043	0.046*	
C15	1.17060 (18)	0.87688 (11)	0.71403 (7)	0.0359 (3)	
H15	1.1805	0.8893	0.7568	0.043*	
C16	1.4524 (3)	0.8232 (2)	0.52666 (11)	0.0807 (9)	
H16A	1.4162	0.7662	0.5247	0.121*	
H16B	1.5648	0.8265	0.5160	0.121*	
H16C	1.3815	0.8560	0.4974	0.121*	
C17	0.45776 (16)	1.02117 (9)	0.80810 (7)	0.0292 (3)	
H17	0.3849	0.9764	0.8089	0.035*	
C18	0.39254 (17)	1.10066 (9)	0.80236 (7)	0.0286 (3)	
C19	0.50145 (18)	1.16809 (10)	0.80272 (8)	0.0333 (3)	
H19	0.4616	1.2225	0.7995	0.040*	
C20	0.66992 (19)	1.15209 (11)	0.80794 (9)	0.0377 (3)	
H20	0.7458	1.1958	0.8086	0.045*	
C21	0.72488 (17)	1.07040 (11)	0.81222 (8)	0.0339 (3)	
H21	0.8386	1.0601	0.8150	0.041*	
C22	0.20678 (17)	1.10968 (10)	0.79763 (8)	0.0324 (3)	
C23	0.99457 (19)	0.91195 (10)	0.92055 (7)	0.0328 (3)	
H23	1.0367	0.9371	0.8862	0.039*	
C24	1.0964 (2)	0.90585 (10)	0.97668 (7)	0.0347 (3)	
C25	1.0320 (3)	0.86721 (12)	1.02707 (8)	0.0469 (4)	
H25	1.0975	0.8605	1.0651	0.056*	
C26	0.8706 (3)	0.83885 (14)	1.02045 (9)	0.0505 (5)	
H26	0.8254	0.8139	1.0542	0.061*	
C27	0.7764 (2)	0.84774 (12)	0.96324 (8)	0.0418 (4)	
H27	0.6673	0.8286	0.9590	0.050*	
C28	1.2709 (2)	0.93956 (12)	0.98526 (8)	0.0414 (4)	

### Atomic displacement parameters (Å^2^)

**Table:**

	*U*^11^	*U*^22^	*U*^33^	*U*^12^	*U*^13^	*U*^23^
Zn1	0.02093 (7)	0.03526 (10)	0.03313 (9)	0.00195 (6)	0.00203 (6)	0.00011 (7)
N1	0.0195 (5)	0.0333 (6)	0.0351 (6)	0.0005 (4)	0.0016 (4)	0.0020 (5)
N2	0.0223 (6)	0.0578 (10)	0.0581 (9)	0.0050 (6)	0.0030 (6)	0.0255 (8)
N3	0.0313 (6)	0.0355 (7)	0.0326 (6)	0.0026 (5)	0.0018 (5)	−0.0002 (5)
N4	0.0339 (7)	0.0644 (10)	0.0413 (8)	−0.0028 (7)	−0.0083 (6)	0.0072 (7)
N5	0.0552 (11)	0.0446 (10)	0.1095 (18)	−0.0141 (9)	0.0262 (11)	0.0041 (11)
N6	0.0287 (7)	0.0872 (13)	0.0372 (7)	−0.0010 (8)	0.0052 (6)	−0.0064 (8)
O1	0.0325 (6)	0.0377 (6)	0.0597 (8)	−0.0058 (5)	0.0043 (5)	0.0075 (6)
O2	0.0329 (6)	0.0584 (8)	0.0607 (8)	−0.0003 (6)	0.0155 (6)	−0.0087 (7)
O3	0.0262 (5)	0.0568 (8)	0.0372 (6)	0.0011 (5)	0.0063 (4)	−0.0029 (5)
O4	0.0200 (5)	0.0701 (9)	0.0529 (7)	−0.0037 (5)	−0.0012 (5)	0.0024 (7)
O5	0.0570 (9)	0.0906 (12)	0.0546 (9)	−0.0181 (9)	−0.0264 (7)	0.0248 (9)
O6	0.0218 (5)	0.0739 (9)	0.0547 (7)	−0.0007 (6)	0.0043 (5)	0.0282 (7)
C1	0.0303 (7)	0.0408 (8)	0.0317 (7)	−0.0034 (6)	0.0027 (6)	−0.0036 (6)
C2	0.0310 (7)	0.0336 (7)	0.0325 (7)	−0.0014 (6)	0.0033 (5)	−0.0029 (6)
C3	0.0384 (8)	0.0342 (8)	0.0539 (10)	0.0043 (7)	0.0092 (7)	−0.0062 (7)
C4	0.0495 (10)	0.0287 (8)	0.0689 (13)	−0.0028 (7)	0.0109 (9)	−0.0041 (8)
C5	0.0397 (9)	0.0375 (9)	0.0517 (10)	−0.0086 (7)	0.0045 (8)	0.0005 (7)
C6	0.0297 (7)	0.0391 (9)	0.0575 (10)	0.0003 (6)	0.0088 (7)	0.0012 (8)
C7	0.0332 (7)	0.0291 (7)	0.0501 (9)	0.0011 (6)	0.0063 (7)	−0.0005 (6)
C8	0.0428 (11)	0.0760 (17)	0.097 (2)	−0.0142 (11)	0.0142 (12)	0.0192 (15)
C9	0.0221 (6)	0.0322 (7)	0.0401 (8)	−0.0007 (5)	0.0031 (5)	0.0007 (6)
C10	0.0203 (5)	0.0355 (7)	0.0332 (7)	−0.0007 (5)	0.0009 (5)	−0.0025 (6)
C11	0.0242 (6)	0.0515 (10)	0.0350 (7)	−0.0034 (6)	−0.0030 (5)	−0.0069 (7)
C12	0.0316 (7)	0.0558 (10)	0.0292 (7)	−0.0009 (7)	−0.0011 (6)	−0.0079 (7)
C13	0.0252 (6)	0.0447 (8)	0.0329 (7)	0.0001 (6)	0.0038 (5)	−0.0012 (6)
C14	0.0224 (6)	0.0578 (11)	0.0346 (7)	−0.0053 (6)	0.0011 (5)	−0.0065 (7)
C15	0.0224 (6)	0.0536 (10)	0.0314 (7)	−0.0048 (6)	0.0012 (5)	−0.0073 (7)
C16	0.0495 (12)	0.149 (3)	0.0462 (12)	0.0039 (16)	0.0179 (10)	−0.0182 (15)
C17	0.0199 (5)	0.0319 (7)	0.0355 (7)	−0.0026 (5)	0.0019 (5)	0.0010 (6)
C18	0.0195 (5)	0.0337 (7)	0.0322 (7)	−0.0002 (5)	0.0014 (5)	0.0029 (5)
C19	0.0253 (6)	0.0315 (7)	0.0423 (8)	−0.0018 (5)	−0.0003 (6)	0.0026 (6)
C20	0.0239 (6)	0.0362 (8)	0.0520 (9)	−0.0088 (6)	−0.0003 (6)	0.0030 (7)
C21	0.0188 (5)	0.0403 (8)	0.0421 (8)	−0.0030 (5)	0.0002 (5)	0.0042 (6)
C22	0.0192 (5)	0.0391 (8)	0.0384 (7)	0.0013 (5)	0.0010 (5)	0.0053 (6)
C23	0.0325 (7)	0.0360 (7)	0.0290 (6)	0.0027 (6)	−0.0009 (5)	0.0006 (6)
C24	0.0382 (8)	0.0352 (7)	0.0293 (7)	0.0044 (6)	−0.0043 (6)	−0.0014 (6)
C25	0.0577 (12)	0.0508 (10)	0.0301 (7)	−0.0012 (9)	−0.0062 (7)	0.0040 (7)
C26	0.0612 (12)	0.0563 (12)	0.0341 (8)	−0.0096 (10)	0.0042 (8)	0.0078 (8)
C27	0.0425 (9)	0.0460 (10)	0.0372 (8)	−0.0031 (8)	0.0055 (7)	0.0016 (7)
C28	0.0390 (8)	0.0473 (10)	0.0352 (8)	0.0023 (7)	−0.0091 (6)	−0.0010 (7)

### Geometric parameters (Å, °)

**Table:**

Zn1—O1	1.9584 (14)	C8—H8C	0.9600
Zn1—O3	1.9210 (14)	C10—C11	1.389 (2)
Zn1—N1	2.0722 (15)	C10—C9	1.492 (2)
Zn1—N3	2.0854 (15)	C11—C12	1.386 (2)
O1—C1	1.284 (2)	C11—H11	0.9300
O2—C1	1.248 (2)	C12—H12	0.9300
O3—C9	1.278 (2)	C13—N6	1.366 (2)
O4—C9	1.2410 (19)	C13—C12	1.408 (2)
O5—C28	1.235 (2)	C14—C13	1.399 (2)
N1—C17	1.3350 (18)	C14—H14	0.9300
N1—C21	1.338 (2)	C15—C14	1.371 (2)
N2—H2A	0.8600	C15—C10	1.398 (2)
N2—H2B	0.8600	C15—H15	0.9300
N3—C23	1.341 (2)	C16—H16A	0.9600
N3—C27	1.343 (2)	C16—H16B	0.9600
N4—C28	1.319 (2)	C16—H16C	0.9600
N4—H4A	0.8600	C17—H17	0.9300
N4—H4B	0.8600	C18—C17	1.380 (2)
N5—C8	1.434 (3)	C18—C22	1.502 (2)
N5—H51	0.90 (3)	C19—C20	1.379 (2)
N6—C16	1.433 (3)	C19—C18	1.394 (2)
N6—H61	0.88 (2)	C19—H19	0.9300
C1—C2	1.477 (2)	C20—H20	0.9300
C2—C7	1.382 (2)	C21—C20	1.383 (3)
C2—C3	1.402 (2)	C21—H21	0.9300
C3—C4	1.374 (3)	C22—O6	1.2333 (19)
C3—H3	0.9300	C22—N2	1.320 (2)
C4—H4	0.9300	C23—C24	1.387 (2)
C5—N5	1.369 (3)	C23—H23	0.9300
C5—C4	1.403 (3)	C24—C25	1.387 (3)
C6—C5	1.404 (3)	C24—C28	1.505 (3)
C6—H6	0.9300	C25—C26	1.376 (3)
C7—C6	1.386 (2)	C25—H25	0.9300
C7—H7	0.9300	C26—H26	0.9300
C8—H8A	0.9600	C27—C26	1.379 (3)
C8—H8B	0.9600	C27—H27	0.9300
			
O1—Zn1—N1	96.85 (6)	C15—C10—C9	120.17 (14)
O1—Zn1—N3	105.85 (6)	C12—C11—C10	121.78 (14)
O3—Zn1—O1	143.41 (6)	C12—C11—H11	119.1
O3—Zn1—N1	105.91 (5)	C10—C11—H11	119.1
O3—Zn1—N3	95.81 (6)	C11—C12—C13	120.21 (15)
N1—Zn1—N3	104.58 (5)	C11—C12—H12	119.9
C1—O1—Zn1	105.71 (11)	C13—C12—H12	119.9
C9—O3—Zn1	117.30 (10)	N6—C13—C14	119.28 (15)
C17—N1—Zn1	119.06 (10)	N6—C13—C12	123.04 (15)
C17—N1—C21	118.13 (14)	C14—C13—C12	117.68 (14)
C21—N1—Zn1	122.69 (10)	C15—C14—C13	121.36 (14)
C22—N2—H2A	120.0	C15—C14—H14	119.3
C22—N2—H2B	120.0	C13—C14—H14	119.3
H2A—N2—H2B	120.0	C14—C15—C10	121.32 (15)
C23—N3—C27	118.54 (15)	C14—C15—H15	119.3
C23—N3—Zn1	120.24 (11)	C10—C15—H15	119.3
C27—N3—Zn1	120.97 (12)	N6—C16—H16A	109.5
C28—N4—H4A	120.0	N6—C16—H16B	109.5
C28—N4—H4B	120.0	H16A—C16—H16B	109.5
H4A—N4—H4B	120.0	N6—C16—H16C	109.5
C5—N5—C8	125.0 (2)	H16A—C16—H16C	109.5
C5—N5—H51	115.8 (18)	H16B—C16—H16C	109.5
C8—N5—H51	119.1 (18)	N1—C17—C18	122.99 (13)
C13—N6—C16	123.28 (17)	N1—C17—H17	118.5
C13—N6—H61	116.0 (16)	C18—C17—H17	118.5
C16—N6—H61	117.5 (16)	C17—C18—C19	118.71 (13)
O2—C1—O1	120.47 (15)	C17—C18—C22	117.73 (13)
O2—C1—C2	121.63 (16)	C19—C18—C22	123.54 (14)
O1—C1—C2	117.86 (14)	C20—C19—C18	118.31 (15)
C7—C2—C3	117.54 (15)	C20—C19—H19	120.8
C7—C2—C1	121.41 (15)	C18—C19—H19	120.8
C3—C2—C1	121.04 (15)	C19—C20—C21	119.31 (14)
C4—C3—C2	121.17 (16)	C19—C20—H20	120.3
C4—C3—H3	119.4	C21—C20—H20	120.3
C2—C3—H3	119.4	N1—C21—C20	122.53 (14)
C3—C4—C5	121.00 (17)	N1—C21—H21	118.7
C3—C4—H4	119.5	C20—C21—H21	118.7
C5—C4—H4	119.5	O6—C22—N2	124.08 (14)
N5—C5—C4	119.96 (18)	O6—C22—C18	119.76 (14)
N5—C5—C6	121.96 (18)	N2—C22—C18	116.16 (14)
C4—C5—C6	118.08 (16)	N3—C23—C24	122.98 (16)
C7—C6—C5	119.76 (16)	N3—C23—H23	118.5
C7—C6—H6	120.1	C24—C23—H23	118.5
C5—C6—H6	120.1	C23—C24—C25	117.63 (17)
C2—C7—C6	122.31 (16)	C23—C24—C28	123.21 (16)
C2—C7—H7	118.8	C25—C24—C28	119.15 (15)
C6—C7—H7	118.8	C26—C25—C24	119.65 (17)
N5—C8—H8A	109.5	C26—C25—H25	120.2
N5—C8—H8B	109.5	C24—C25—H25	120.2
H8A—C8—H8B	109.5	C25—C26—C27	119.34 (18)
N5—C8—H8C	109.5	C25—C26—H26	120.3
H8A—C8—H8C	109.5	C27—C26—H26	120.3
H8B—C8—H8C	109.5	N3—C27—C26	121.83 (18)
O4—C9—O3	123.17 (15)	N3—C27—H27	119.1
O4—C9—C10	121.35 (15)	C26—C27—H27	119.1
O3—C9—C10	115.48 (13)	O5—C28—N4	122.70 (18)
C11—C10—C15	117.64 (14)	O5—C28—C24	119.20 (18)
C11—C10—C9	122.18 (13)	N4—C28—C24	118.09 (15)
			
O3—Zn1—O1—C1	46.26 (16)	C4—C5—N5—C8	176.6 (2)
N1—Zn1—O1—C1	174.95 (11)	C6—C5—N5—C8	−3.9 (4)
N3—Zn1—O1—C1	−77.74 (11)	C7—C6—C5—N5	178.0 (2)
O1—Zn1—O3—C9	46.46 (17)	C7—C6—C5—C4	−2.6 (3)
N1—Zn1—O3—C9	−79.85 (13)	C2—C7—C6—C5	−0.8 (3)
N3—Zn1—O3—C9	173.16 (12)	C11—C10—C9—O4	−6.2 (3)
O3—Zn1—N1—C17	143.78 (11)	C15—C10—C9—O4	173.48 (16)
O1—Zn1—N1—C17	−7.29 (12)	C11—C10—C9—O3	173.75 (16)
N3—Zn1—N1—C17	−115.68 (12)	C15—C10—C9—O3	−6.6 (2)
O3—Zn1—N1—C21	−40.14 (14)	C15—C10—C11—C12	0.3 (3)
O1—Zn1—N1—C21	168.79 (13)	C9—C10—C11—C12	179.99 (17)
N3—Zn1—N1—C21	60.41 (14)	C10—C11—C12—C13	−0.7 (3)
O3—Zn1—N3—C23	28.53 (13)	C14—C13—N6—C16	170.4 (2)
O1—Zn1—N3—C23	178.74 (12)	C12—C13—N6—C16	−9.6 (3)
N1—Zn1—N3—C23	−79.60 (13)	N6—C13—C12—C11	−179.86 (19)
O3—Zn1—N3—C27	−145.61 (14)	C14—C13—C12—C11	0.2 (3)
O1—Zn1—N3—C27	4.61 (15)	C15—C14—C13—N6	−179.25 (19)
N1—Zn1—N3—C27	106.26 (14)	C15—C14—C13—C12	0.7 (3)
Zn1—O1—C1—O2	−3.24 (19)	C14—C15—C10—C11	0.6 (3)
Zn1—O1—C1—C2	174.63 (11)	C14—C15—C10—C9	−179.10 (16)
Zn1—O3—C9—O4	−3.2 (2)	C10—C15—C14—C13	−1.1 (3)
Zn1—O3—C9—C10	176.82 (10)	C19—C18—C17—N1	−1.6 (2)
C21—N1—C17—C18	1.0 (2)	C22—C18—C17—N1	179.83 (14)
Zn1—N1—C17—C18	177.25 (12)	C17—C18—C22—O6	43.7 (2)
C17—N1—C21—C20	0.4 (2)	C19—C18—C22—O6	−134.76 (18)
Zn1—N1—C21—C20	−175.73 (13)	C17—C18—C22—N2	−135.66 (17)
C27—N3—C23—C24	0.8 (2)	C19—C18—C22—N2	45.9 (2)
Zn1—N3—C23—C24	−173.51 (12)	C20—C19—C18—C17	0.9 (2)
C23—N3—C27—C26	−1.3 (3)	C20—C19—C18—C22	179.33 (15)
Zn1—N3—C27—C26	172.97 (15)	C18—C19—C20—C21	0.4 (3)
O2—C1—C2—C7	172.14 (16)	N1—C21—C20—C19	−1.1 (3)
O1—C1—C2—C7	−5.7 (2)	N3—C23—C24—C25	0.8 (3)
O2—C1—C2—C3	−6.5 (2)	N3—C23—C24—C28	−178.69 (16)
O1—C1—C2—C3	175.65 (16)	C23—C24—C25—C26	−1.8 (3)
C7—C2—C3—C4	−1.3 (3)	C28—C24—C25—C26	177.67 (18)
C1—C2—C3—C4	177.42 (17)	C23—C24—C28—O5	−169.02 (19)
C3—C2—C7—C6	2.7 (3)	C25—C24—C28—O5	11.5 (3)
C1—C2—C7—C6	−176.01 (16)	C23—C24—C28—N4	11.6 (3)
C2—C3—C4—C5	−2.0 (3)	C25—C24—C28—N4	−167.84 (18)
N5—C5—C4—C3	−176.6 (2)	C24—C25—C26—C27	1.4 (3)
C6—C5—C4—C3	3.9 (3)	N3—C27—C26—C25	0.2 (3)

### Hydrogen-bond geometry (Å, °)

**Table:**

*D*—H···*A*	*D*—H	H···*A*	*D*···*A*	*D*—H···*A*
N2—H2B···O2^i^	0.86	2.00	2.839 (3)	166
N4—H4B···O6^ii^	0.86	2.12	2.950 (3)	161
N6—H61···O4^ii^	0.88 (2)	2.10 (2)	2.955 (3)	166 (2)

Symmetry codes: (i) −*x*+1, *y*+1/2, −*z*+3/2; (ii) *x*+1, *y*, *z*.
